# Determinants of activity and efficacy of anti-PD1/PD-L1 therapy in patients with advanced solid tumors recruited in a clinical trials unit: a longitudinal prospective biomarker-based study

**DOI:** 10.1007/s00262-022-03360-9

**Published:** 2023-01-10

**Authors:** Javier García-Corbacho, Alberto Indacochea, Azucena E. González Navarro, Iván Victoria, Débora Moreno, David Pesántez, Laura Angelats, Andrea Modrego-Sanchez, Esther Sanfeliu, Oleguer Castillo, Paula Blasco, Laura Mezquita, Nuria Viñolas, Miquel Nogué, Patricia Galván, Barbara Adamo, Neus Basté, Tamara Sauri, Manel Juan, Aleix Prat, Francesco Schettini

**Affiliations:** 1 Medical Oncology Department, Hospital Clinic of Barcelona, Barcelona, Spain; 2 Medical Oncology Department (UGCI), Virgen de La Victoria and Regional University Hospital / IBIMA, Málaga, Spain; 3 Medical Oncology Department, Hospital General of Granollers, Barcelona, Spain; 4 Immunology Department, Hospital Clinic of Barcelona, Barcelona, Spain; 5 Translational Genomics and Targeted Therapies in Solid Tumours Group, August Pi i Sunyer Biomedical Research Institute (IDIBAPS), Barcelona, Spain; 6 Medical Oncology Department, University Hospital, 12 de Octubre, Madrid, Spain; 7 Pathology Department, Diagnostic Biomedical Center, Hospital Clinic of Barcelona, Barcelona, Spain; 8 Departament de Medicina, Facultat de Medicina i Ciències de la Salut, Universitat de Barcelona, c. Casanova, 143, 08036 Barcelona, Spain

**Keywords:** Immunotherapy, Immune checkpoint inhibitors, PD-L1, PD1, Solid tumors

## Abstract

Immune-checkpoint inhibitors (ICI) have revolutionized the therapeutic landscape of cancer. However, optimal patient selection is still an unmet need. One-hundred-forty-six patients with metastatic cancer candidates to ICI at the Hospital Clinic of Barcelona Clinical Trials Unit were prospectively recruited in this observational study. Blood samples were collected at different timepoints, baseline LIPI score calculated and pre-ICI archived tissues retrieved to evaluate PD-L1, tumor-infiltrating lymphocytes (TILs) and PD1 mRNA levels. Tumor assessments were centrally reviewed by RECIST 1.1 criteria. Associations with overall response rates (ORR), durable clinical benefit (DCB), progression-free survival (PFS) and overall survival (OS) were performed with univariable/multivariable logistic and Cox regressions, where appropriate. At a median follow-up of 26.9 months, median PFS and OS were 2.7 and 12.9 months. Response rates were 17.8% with duration of response (DOR) of 4.4 months. LIPI score was independently associated with PFS (*p* = 0.025) and OS (*p* < 0.001). Immunotherapy-naïve status was independently associated with better PFS (*p* = 0.005). Time-to-best response (TTBR) and ORR (*p* < 0.001 both) were associated with better OS at univariate analysis. PFS and DOR were moderately correlated with OS (*p* < 0.001 both). A PD-L1 10% cut-off detected worse/best responders in terms of ORR (univariate p = 0.011, multivariate *p* = 0.028) and DCB (univariate *p* = 0.043). PD1 mRNA levels were strikingly associated to complete responses (*p* = 0.021). To resume, in our prospective observational pan-cancer study, baseline LIPI score, immunotherapy-naïve status, cancer type and RT before starting ICI were the most relevant clinical factors independently correlated with immunotherapy outcomes. Longer TTBR seemed to associate with better survival, while PD1 mRNA and PD-L1 protein levels might be tumor-agnostic predictive factors of response to ICI and should be furtherly explored.

**Supplementary Information:**

The online version contains supplementary material available at 10.1007/s00262-022-03360-9.

## Introduction

In the last decade, immunotherapy with immune-checkpoint inhibitors (ICI) has revolutionized the therapeutic landscape of many solid tumors. ICI-based therapeutic approach is based on the disruption of the activity of several immune system inhibitory mechanisms, so to unleash a potent immune response directed toward the tumor [1]. The majority of currently approved ICI act through the inhibition of the PD1/PD-L1 axis [2]. As of today, anti-PD1 (e.g., pembrolizumab, nivolumab) and anti-PD-L1 (e.g., atezolizumab, durvalumab) monoclonal antibodies (mAb) have become some of the most widely prescribed anticancer therapies and are recommended, in monotherapy or combination with other ICI or chemotherapy (CT), in a broad spectrum of cancer types [1]. However, the degree of benefit is different according to the cancer type and within each tumor type, and only a limited proportion of patients seem to benefit [3].

The only predictive biomarkers of response that can be used in clinical practice are the assessment of PD-L1 levels by immunohistochemistry (IHC), micro-satellite instability (MSI) and tumor mutational burden (TMB), though the latter only in the USA [4–7]. However, they have been variably successful in predicting responders according to different cancers and their use is limited to specific contexts [4–6]. The outcome of ICI therapy has also been linked to the quality and magnitude of tumor-infiltrating lymphocytes (TILs)’ responses within the tumor microenvironment, though without current clinical applicability [8]. Additionally, the optimal metastatic therapeutic setting (earlier or further lines), the efficacy in immune-pretreated patients, the effects of exposure to immediately previous or concurrent radiotherapy (RT), and the optimal duration of treatment remain questions unanswered. To note, the impact of systemic corticosteroids and exposure to antibiotic (ATB) therapy on response to ICI are another major concern, with only few and/or conflicting data being published so far [9–18]. Finally, easy-to-detect and relatively low cost prognostic predictors able to stratify patients for either ICI clinical trial inclusion or better tailoring of the treatment strategy are urgently needed and the LIPI score, based on a relative neutrophil count and LDH is a promising one, which merits further validation in a pan-cancer setting [19, 20].

The Bioimmunoblood project is a prospective observational study which is currently ongoing at the Clinical Trials Unit of the Hospital Clinic of Barcelona (HCB) Medical Oncology Department. Within this project we aim at characterizing the patterns of response to anti-PD1 and anti-PD-L1 ICI in metastatic solid tumors and exploring patients’ clinicopathological, molecular and blood features that can be useful to improve the selection of candidates for this relatively novel therapeutic approach. Here we report the main clinical results, while extensive molecular characterization and blood biomarker study are currently ongoing.

## Materials and methods

### Study design and participants

**Fig. 1:**
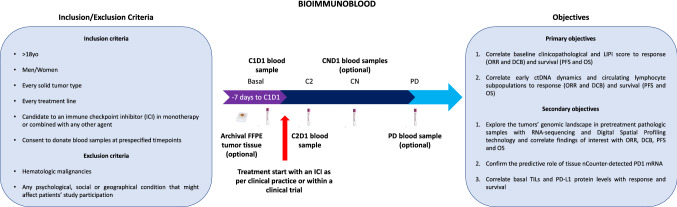
Bioimmunoblood study design. *C* cycle, *D* day, *FFPE* fresh-frozen paraffin-embedded, *ICI* immune-checkpoint inhibitors, *ORR* overall response rate, *DCB* durable clinical benefit, *PFS* progression-free survival, *OS* overall survival, *TILs* tumor-infiltrating lymphocytes, *ctDNA* circulating tumor DNA, *PD* progressive disease, *yo* years old

To enter the Bioimmunoblood study, eligible patients had to be diagnosed of metastatic solid tumor and about to start a treatment with an ICI in a clinical trial. Full inclusion/exclusion criteria are reported in Fig. 1.

We considered evaluable for this analysis all participants treated with an anti-PD1 or anti-PD-L1 ICI with radiological data available for an independent assessment of tumor responses according to RECIST 1.1 criteria [21]. Patients with available baseline imaging experiencing a rapid progression leading to death, hence with no available radiologic reassessment, were also included.

### Procedures

A blood sample was collected from each patient at the first day of cycle 1 (C1D1) and 2 (C2D1) prior to receive the therapy and at each radiological evaluation of response until progression. For this analysis only basal samples were considered. Blood chemistry tests were carried out, including the evaluation of albumin, hemoglobin (Hb), LDH and standard leukocyte populations. The lung immune prognostic index (LIPI) score was also calculated [22]. Treatments and follow-up procedures were decided outside of this study according to study protocol, since patients received ICI in interventional clinical trials. All data were retrieved from electronic patient charts. In case of availability and explicit patient consent, archived tumor sections from the primary or the latest available metastatic biopsy before starting ICI were collected. An expert pathologist from the HCB (ES) carried out an assessment of TILs according to the methodology proposed by the International Immuno-Oncology Biomarkers Working Group [23]. PD1 mRNA expression was evaluated using the Nanostring nCounter^®^ platform as we elsewhere described [24]. PD-L1 was assessed according to the HCB clinical practice and using the anti-PD-L1 mouse monoclonal antibody 22C3 (Dako), following manufacturer’s recommendation [25, 26] (Supplementary materials).

### Study endpoints and outcomes

There was no prespecified sample size because of the exploratory nature of this study. The accrual was terminated after 4 years, and the clinical data cut-off was established when a minimum follow-up including at least one reassessment of the disease for every included patient was reached.

This first analysis was intended to correlate baseline clinicopathological factors to response, in terms of overall response rate (ORR) and durable clinical benefit (DCB), and survival, in terms of progression-free survival (PFS) and overall survival (OS) (Primary Objective 1, Fig. 1). The primary features of interest were treatment line at which an anti-PD1 or PD-L1 ICI is delivered (1^st^ vs. subsequent lines), patients’ immune-naïve status (yes vs. no), the regimen type (ICI monotherapy vs. ICI-based combination), the ICI target (anti-PD1 vs. anti-PD-L1), having received RT, systemic ATB or corticosteroids (> 10 mg prednisone equivalent dose) within 30 days before, or during ICI treatment, as well as cancer type according to the following groups: NSCLC, genitourinary (GU) tumors, gastrointestinal (GI) tumors, breast cancer/gynecological tumors, other rarer tumors. The effect on OS for the time-to-best response (TTBR) and duration of response (DOR) in patients achieving at least a stable disease (SD), was investigated, as well. The prognostic value of the LIPI score in terms of PFS and OS in a pan-cancer context was also assessed.

Further objectives of this first report were to explore TILs, PD-L1 protein and PD1 mRNA impact on ORR, DCB, PFS and OS in patients treated with anti-PD1/PD-L1 ICI (Secondary Objectives 2–3, Fig. 1).

The evaluation of response for the purpose of this study were performed in accordance to RECIST 1.1 criteria [21]. Best responses (BR) were classified as SD, progressive disease (PD), complete (CR) or partial response (PR) independently by the same expert (JGC) from the Clinical Trials Unit of the HCB [21]. For the ORR assessment we considered all patients achieving CR + PR as BR, while for DCB we included all patients achieving CR + PR + SD retained at 6 months as BR.

### Statistical analysis

Multiple χ^2^ tests and one-way ANOVA were used to calculate differences among poor, best and non-responders with respect to categorical and continuous variables of interest, respectively. For the purpose of this study, we considered as poor responders all patients that achieved SD as their BR, while best responders were those achieving PR or CR as their BR and non-responders were represented by patients with PD as BR. Correlations between continuous variables were evaluated with Pearson’s r. Univariate and multivariable logistic regression analyses were performed to investigate the association between PD1 mRNA abundance with tumor response. Odds ratios (OR) with 95% confidence intervals (CI) were used as measure of association with ORR and DCB. The maximally selected rank statistics (MSRS) method was adopted to identify an exploratory optimal cut-off for PD1 mRNA, TILs and PD-L1 protein, considering PFS as the time-dependent endpoint [27]. Survival curves were estimated by the Kaplan–Meier method and differences between curves were evaluated by the log-rank test. Cox regression models were applied to estimate univariate and multivariate hazard ratios (HR) with their 95% CI to explore the association among clinicopathological/biological variables, TTBR, DOR, PFS and OS. For the primary endpoint of PFS, the proportional hazard assumption for the univariate and multivariate Cox regression models was previously tested using correlation coefficients between transformed survival times and scaled Schoenfeld residuals and further checked with the smoothed plots of Schoenfeld residuals [28]. The clinical data cut-off date for this analysis was 25 August 2021. Patients alive were censored at the date of the last follow-up.

A two-sided alfa error of 0.5 was considered for statistical significance. Considering the observational and exploratory nature of the study, we decided not to take into account the multiplicity issue [29, 30]. All statistical analyses were carried out using R Studio vers.1.0.153 (PBC, Boston, MA) and SPSS vers 24.0 (IBM SPSS Statistics, Armonk, NY: IBM Corp) for MacOSX. Full methods are reported in Supplementary materials.

## Results

**Fig. 2:**
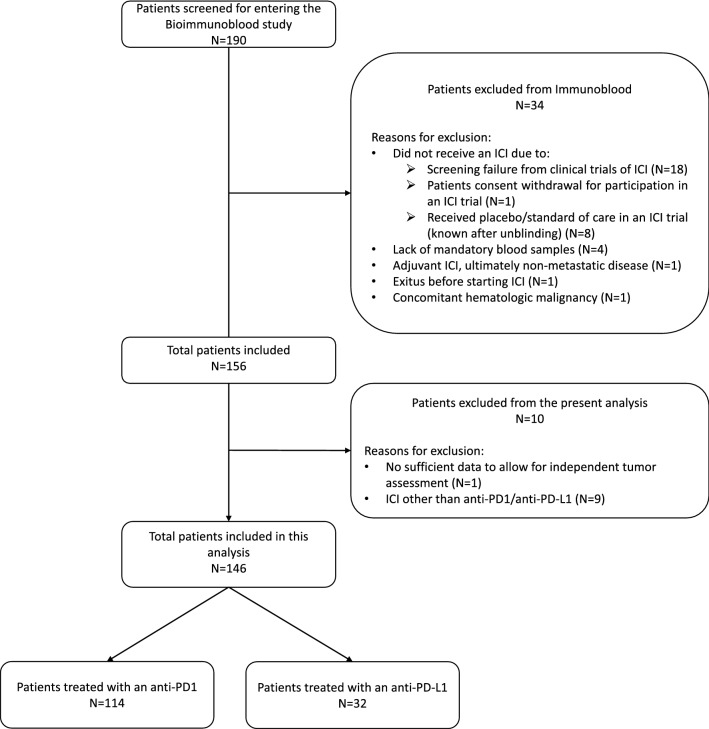
STROBE flowchart. *ICI* immune-checkpoint inhibitors

Between May 2017 and June 2021, 156 patients entered the study and 146 received an anti-PD1/anti-PD-L1-based treatment. The selection process for the purpose of this analysis is resumed in Fig. 2.

The median follow-up at the data cut-off (31/08/2021) was 26.9 months (95% CI: 13.1–31.7). All patients and tumors characteristics are detailed in Table 1.

**Table 1:**
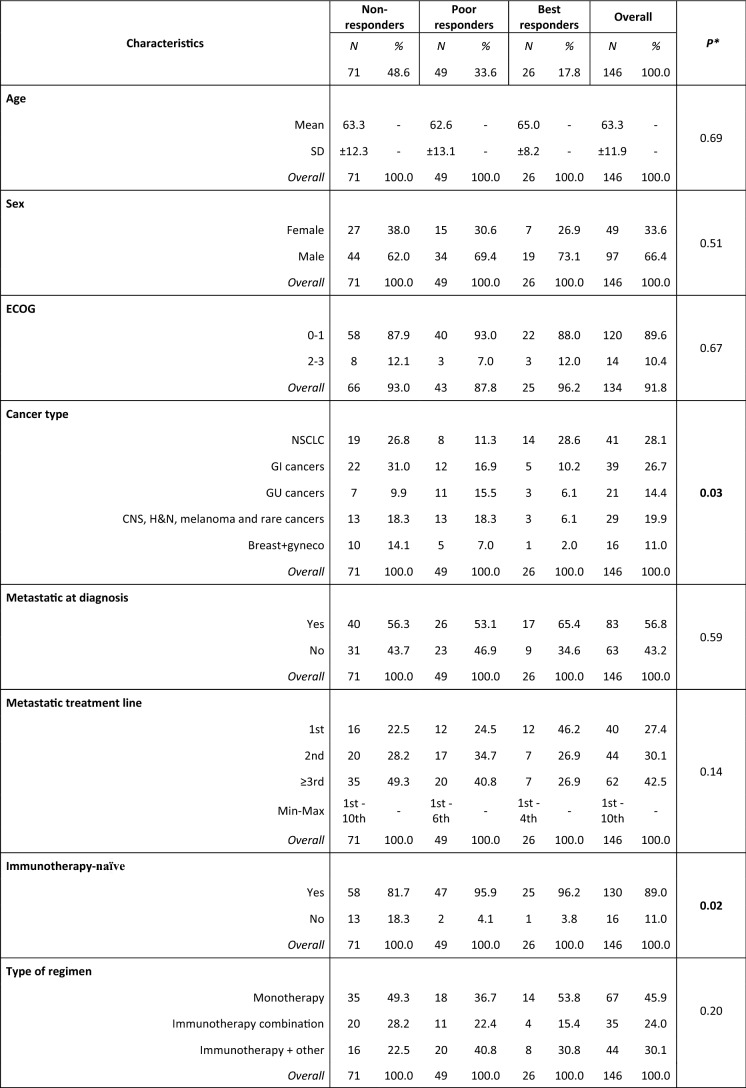

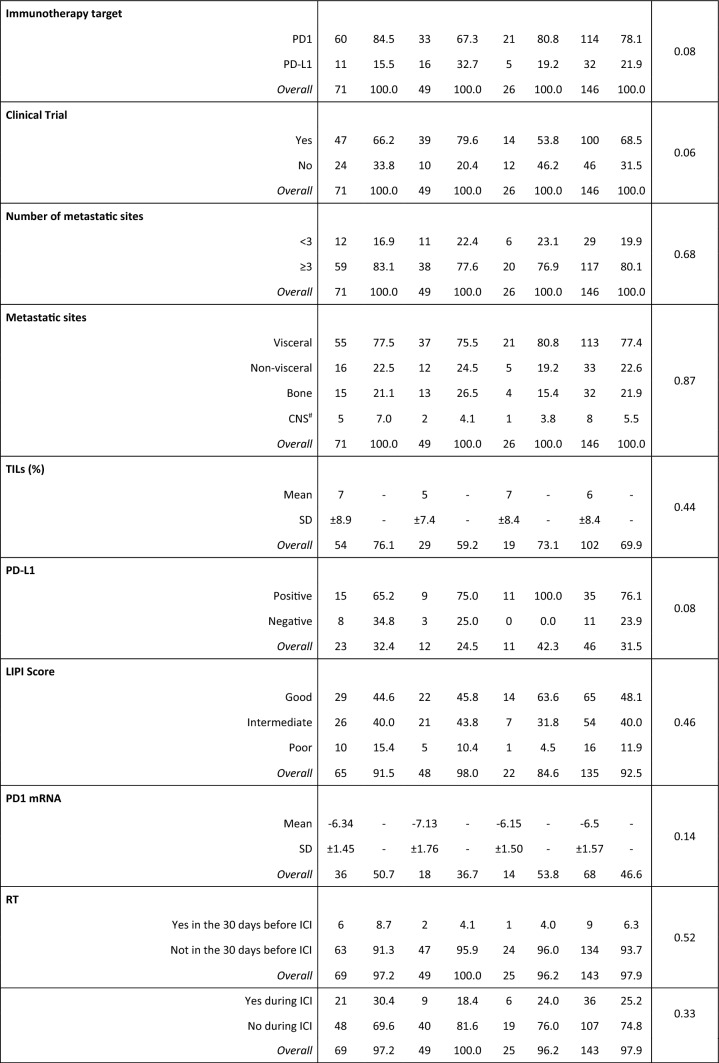

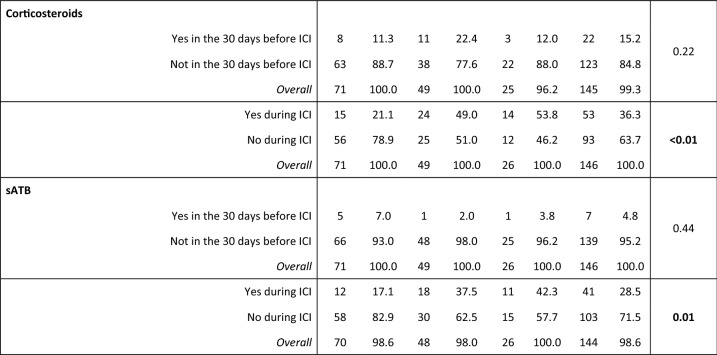
Population characteristics

*Non-responders* progressive disease as best response, *Poor responders* stable disease as best response, *Best responders* complete response or partial response as best response, *SD* standard deviation, *CNS* central nervous system, *ICI* immune-checkpoint inhibitors, *TILs* tumor-infiltrating lymphocytes, *sATB* systemic antibiotics, *RT* radiotherapy, *GI* gastrointestinal, including colorectal, gastric, esophageal, pancreatic cancer and cholangiocarcinoma, *GU* genitourinary, including kidney, bladder urothelial and prostate cancer, *Gyneco* gynecological, including ovarian and cervix cancer, CNS tumors includes only glioblastoma, *H&N* head and neck tumors; rare tumors include sarcomas, thymic and suprarenal carcinomas, *NSCLC* non-small cell lung cancer, *χ^*2*^ test for differences in proportions and unpaired Student’s t test for differences in means, # primary CNS tumors excluded

A summary of activity and efficacy outcomes is reported in Table 2.

**Table 2:**
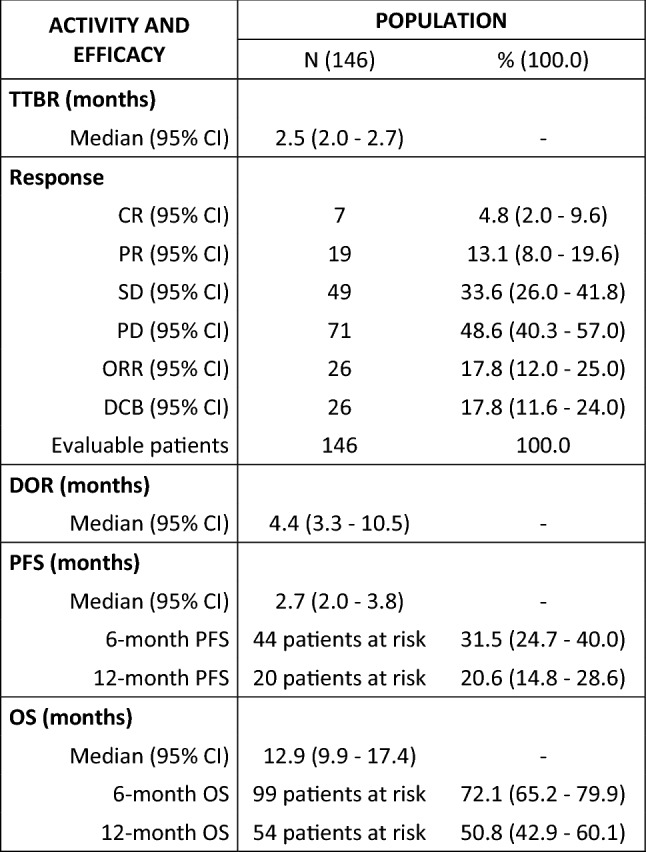
Overall ICI activity and efficacy

*TTBR* time-to-best response, *DOR* duration of response, *PFS* progression-free survival, *OS* overall survival, *CI* confidence interval, *CR* complete response, *PR* partial response, *SD* stable disease, *PD* progressive disease, *ORR* overall response rate, *DCB* durable clinical benefit

### Progression-free survival

At the time of data cut-off, 120 PFS events had occurred and median PFS was 2.7 months (95% CI 2.0–3.8) (Supplementary Fig. 1 and Table 2).

**Fig. 3:**
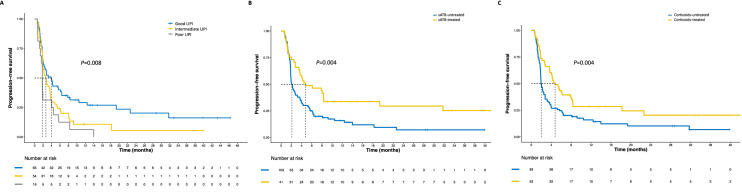
Progression-free survival curves according to significant population characteristics. *A* PFS according to LIPI score, *B* PFS according to sATB administration during ICI treatment, *C* PFS according to systemic corticosteroids administration during ICI treatment, *PFS* progression-free survival, *sATB* systemic antibiotics, *ICI* immune-checkpoint inhibitors

Cancer site showed a significant association with PFS at the univariate analysis (*p* = 0.007) (Fig. 3), with NSCLC patients treated with ICI being significantly favored over patients with GI tumors (*p* = 0.011), breast cancer and other gynecological malignancies (*p* = 0.012), melanoma, H&N tumors and other rare malignancies (*p* = 0.003) but not genitourinary cancers (*p* = 0.628). Patients treated in first-line showed better PFS than patients treated in later lines (*p* = 0.037) (Fig. 3), and the later the line, the worse the outcome (*p* = 0.001). LIPI score was significantly associated with PFS (*p* = 0.008) (Fig. 3), with intermediate (*p* = 0.035) and poor scores (*p* = 0.005) associated with worse PFS than good scores. Immuno-naïve status, systemic ATB and corticosteroids during ICI were also associated with significant PFS improvement (*p* = 0.001, *p* = 0.004 and *p* = 0.004, respectively) (Fig. 3). No other clinical or hematological factors were associated with PFS (full results in Supplementary Table 1).

At the multivariate analysis, only immunotherapy-naïve status (*p* = 0.005) and LIPI score (*p* = 0.025) were associated with PFS independently from each other, cancer site, treatment line, ATB, corticosteroids and previous RT (Table 3).

**Table 3:**
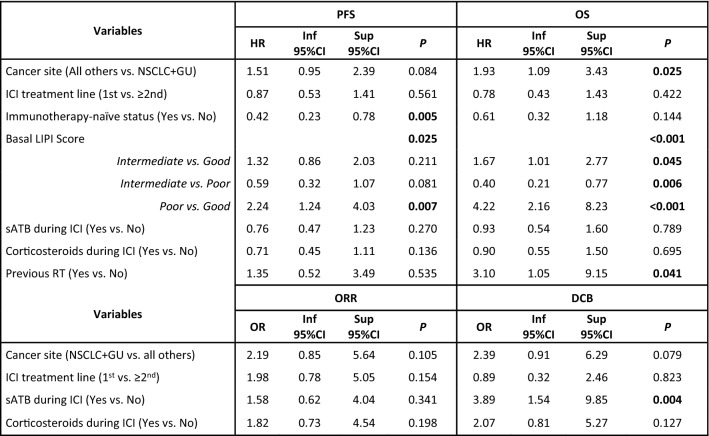
Multivariate survival analyses

*HR* hazard ratio, *OR* odds ratio, *Inf* inferior, *Sup* superior, *PFS* progression-free survival, *OS* overall survival, *ORR* overall response rate, *DCB* durable clinical benefit, *ICI* immune-checkpoint inhibitor, *CR* complete response, *PR* partial response, *SD* stable disease, *PD* progressive disease, *NSCLC* non-small cell lung cancer, *GU* genitourinary, *sATB* systemic antibiotics, *RT* radiotherapy

Significant *p* values are reported in bold

PFS showed a positive moderate correlation with OS: *r* = 0.75, *p* < 0.001.

### Activity

The median TTBR was 2.5 months (95%CI 2.0–2.7) (Supplementary Fig. 1), with an ORR of 17.8% (95%CI 12.0–25.0%) (Table 2). Excluding patients who experienced a PD as best response, the median DOR was 4.4 months (95%CI 3.3–10.5) (Supplementary Fig. 1), with 17.8% (95%CI 11.6–24.0%) patients experiencing a CR, PR or SD lasting ≥ 6 months (Table 2). The DOR showed a positive moderate correlation with OS (*r* = 0.60, *p* < 0.001).

**Fig. 4:**
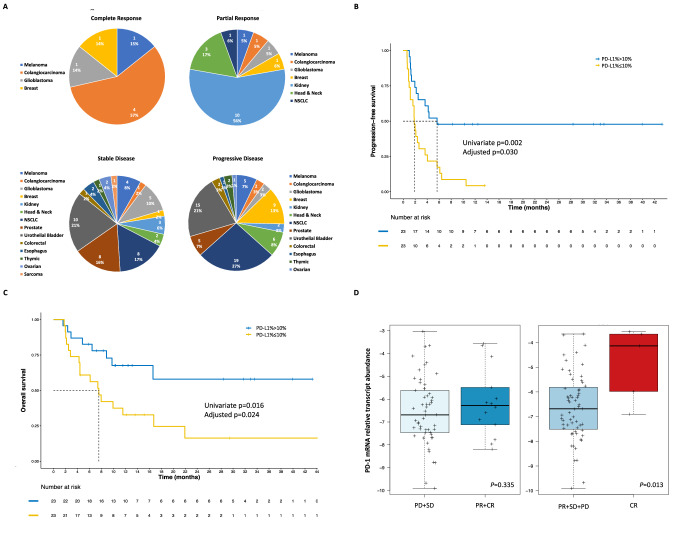
PD-L1 protein and PD1 mRNA levels’ main associations with outcomes and best responses according to tumor site. *A* Best response according to tumor site, *B* Progression-free survival KM curves according to a PD-L1 cut-off selected with the Maximally Selected Rank Statistics method, *C* Overall survival KM curves according to the selected PD-L1 cut-off, *D* PD1 mRNA levels in patients achieving an objective response versus patient not achieving an objective response in the left box plot and PD1 mRNA levels in patients achieving a complete response vs. patients not achieving a complete response in the right box plot, *PFS* Progression-free survival, *OS* Overall survival, *KM* Kaplan–Meier, *CR* complete response, *PR* partial response, *SD* stable disease, *PD* progressive disease, *p* values in box plots are referred to Student’s t tests for differences in mean PD1 mRNA levels

Cancer site appeared to be correlated with the achievement of ORR (*p* = 0.044), with NSCLC and GU tumors being associated with better ORR, compared to other cancers (*p* = 0.011) (Fig. 4, Supplementary Fig. 2).

First-line ICI appeared to be associated with stronger responses, compared to later lines (*p* = 0.021) (Supplementary Fig. 2). Systemic ATB during ICI were associated with increased DCB (*p* = 0.001) but not ORR (*p* = 0.089). Notably, systemic corticosteroids administered during ICI were associated with significantly better ORR (*p* = 0.044) and DCB (*p* = 0.015). There were no other significant associations with ORR and DCB (Supplementary Table 2).

Overall results were not significant at the multivariate analysis for ORR (Table 3). Conversely, sATB during ICI were independently associated with more favorable DCB (*p* = 0.004) and a trend for better DCB was observed for NSCLC and GU tumors versus all others (*p* = 0.079) (Table 3).

### Overall survival

**Fig. 5:**
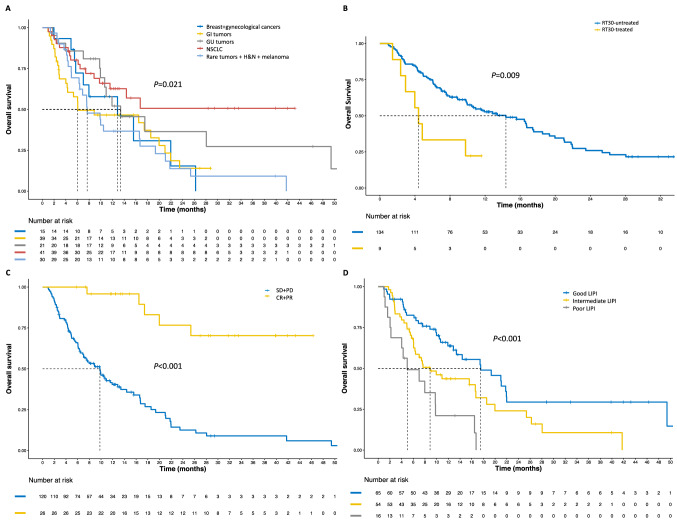
Overall survival curves according to significant population characteristics. *A* OS according to cancer site, *B* OS according to treatment line, *C* OS according to best responses, *D* OS according to LIPI score, *OS* overall survival, *NSCLC* non-small cell lung cancer, *H&N* head and neck tumors, *GI* gastrointestinal, *GU* genitourinary, *SD* stable disease, *PD* progressive disease, *CR* complete responses; *PR* partial responses, *RT30* radiotherapy received within 30 days from ICI start

At the time of data cut-off, 91 deaths had occurred, and median OS was of 12.9 months (95%CI 9.9–17.4) (Supplementary Fig. 1 and Table 2). Similarly to PFS, tumor site, number of treatment lines and LIPI score were significantly associated with OS (*p* = 0.021, *p* = 0.037 and *p* < 0.001, respectively) (Fig. 5). When RT was administered within 30 days before ICI treatment start, a significantly worse OS was observed (*p* = 0.009). Patients achieving an objective response were also prognostically favored over patients achieving SD or PD as their best response (*p* < 0.001) (Fig. 5), with better prognosis for longer TTBR (*p* < 0.001). No other clinical or hematological factors were associated with OS (Supplementary Table 1).

At the multivariate analysis, the independent prognostic value of the LIPI score (*p* < 0.001) was confirmed, along with a detrimental effect for RT received within 30 days before ICI was confirmed (*p* = 0.041), as well. Also, compared to NSCLC and GU tumors, all other cancers showed significantly worse OS (*p* = 0.025) (Table 3).

### Tissue biomarkers exploratory analysis

PD-L1 protein expression, TILs levels and PD1 mRNA levels could be assessed for 46 (31.5%), 102 (69.9%) and 68 (46.6%) patients, respectively.

Increasing protein levels of PD-L1 were found to be associated with slightly better PFS (HR: 0.987, 95%CI 0.978–0.995, p = 0.003). The MSRS method was then applied to detect a potential cut-off of PD-L1 expression to identify patients at better/worse prognosis in terms of PFS. An optimal cut-off of 10% could identify patients with significantly different PFS (≤ 10% vs. > 10% HR: 3.12, 95%CI 1.53–6.36, *p* = 0.002), also when adjusting for cancer site (*p* = 0.030) (Fig. 4, Supplementary Table 1). Additionally, higher levels of PD-L1 were associated with significantly better ORR (OR: 1.03, 95%CI 1.01–1.05, *p* = 0.007) and DCB (OR: 1.03, 95%CI 1.00–1.05, *p* = 0.028). The previously established 10% cut-off was able to distinguish between best/worst responders in terms of ORR (*p* = 0.011) and DCB (*p* = 0.043) at univariate analysis, as well (Supplementary Table 2). When adjusting for cancer site, the cut-off retained its significance in terms of ORR (OR: 11.67, 95%CI 1.30–104.82, *p* = 0.028). Finally, the PD-L1 cut-off was also able to distinguish between patients with worse/better OS at univariate analysis (HR: 2.83, 95%CI 1.22–6.57, *p* = 0.016) and when adjusting for cancer site (*p* = 0.024) (Fig. 4, Supplementary Table 1).

Both TILs and PD1 mRNA levels were not significantly associated to PFS (*p* = 0.730 and *p* = 0.682, respectively), ORR (*p* = 0.742 and *p* = 0.331, respectively), DCB (*p* = 0.870 and *p* = 0.352, respectively) and OS (*p* = 0.509 and *p* = 0.208, respectively) (Supplementary Tables 1 and 2). However, PD1 mRNA levels were strikingly associated to the achievement of CR (Fig. 4), compared to all other responses (OR: 2.35, 95%CI 1.14–4.87, *p* = 0.021) and achieving an objective response was associated to better OS, as previously reported (HR: 0.12, 95%CI 0.05–0.30, *p* < 0.001).

## Discussion

Here we assessed the correlation among many clinicopathological and biological factors with activity and efficacy endpoint of ICI treatment, so to identify an easily detectable profile of the patients that might gain the most benefit out of anti-PD1/PD-L1 immunotherapy. Overall, baseline LIPI score, immunotherapy-naïve status, cancer type and RT before starting ICI were the most relevant clinical factors independently correlated with immunotherapy outcomes. Longer TTBR seem to associate with better survival, suggesting the need for not interrupting ICI therapy unless required for tumor progression, tolerability issues or patient’s preference. We also observed that PD1 mRNA and PD-L1 protein levels might be tumor-agnostic predictive factors of response to ICI.

We confirmed that roughly 18% of patients treated with anti-PD1/PD-L1 ICI experienced a durable clinical response of at least 6 months, including SD. In patients achieving disease control, the DOR moderately correlated with OS and the longer the DOR, the better the OS. Importantly, the TTBR also seemed to be positively correlated with OS. Considering that no specific factors are currently able to prospectively predict the best response the patient will achieve, nor for how long it will last, these results suggest that anti-PD1/PD-L1 ICI might be preferably discontinued at tumor progression or unacceptable toxicity, justifying maintenance/durable treatment strategies.

Unfortunately, only 17.8% patients were able to achieve an objective response (CR or PR), and the type of response was associated with OS, with patients achieving CR or PR as best response experiencing an 88% reduction in the risk of death, compared to patients not achieving an objective response. In this perspective, although the number of cases with tumor tissue available for mRNA detection was too low for introducing the variable in the multivariate logistic regression models, we confirmed the capability of PD1 mRNA to identify patients more likely to achieve an objective response, CR above all (Fig. 4), as our group previously demonstrated [24]. Interestingly, while TILs seemed not to correlate with response and survival outcomes in a pan-cancer context, PD-L1% was positively associated with a slightly higher likelihood of achieving an objective response (OR: 1.03) and a 1% reduction in the risk of progression or death for each unitary increase. Additionally, a cut-off of 10% appeared to be optimal in discriminating between patients at higher likelihood of achieving an objective and durable response and at lower risk of progression or death, similarly to what observed for example, with pembrolizumab in metastatic triple negative breast cancer [31]. Nevertheless, a larger casuistry is required to confirm the result independently from other variables and across cancer types, along with a uniform assessment of PD-L1 throughout cancer types.

We investigated in our study the role of palliative RT administered right before or during anti-PD1/PD-L1 ICI therapy. It has been considered that RT might potentially contribute to determine a stronger systemic immune response (i.e., the abscopal effect) via immunogenic cell death and antigen release, thus enhancing the efficacy of ICI [32, 33]. However, in our cohort, RT administered during ICI was not associated to PFS, OS or tumor responses. Surprisingly, RT administered within 30 days from ICI treatment start was associated with worse OS, independently from all other clinicopathological factors considered. We have no current explanation for this observation and only 9 patients had received palliative RT immediately before ICI start, making this finding difficult to generalize. Conversely, in line with other findings [34, 35], we did not observe any abscopal effect, providing more evidence to debunk a widely postulated, yet scarcely objectivized phenomena [33].

Recently, Pinato et al. showed that systemic ATB administered prior to, but not during ICI monotherapy, are associated with a worse treatment response and OS in solid tumors [9], while ATB treatment in general seems not to impact on chemo-immunotherapy outcomes [10]. In our cohort, only ATB during, but not previous to anti-PD1/PD-L1 treatment, were associated with better PFS (univariate analysis) and DCB (univariate and multivariate analysis). To note, considering the very low number of patients (n = 7) that received ATB prior to ICI, we cannot completely exclude that an ATB-induced gut microbial dysbiosis might impair ICI efficacy. At the same time, we had no sign of detrimental effect during ICI-based therapy in a wider number of patients (n = 41), in line with recent evidences [9, 10], with a significant and independent association to DCB which merits further investigation.

Whether systemic corticosteroids, due to their immunosuppressive effect, might impair or not ICI when administered right before or during treatment is another matter of debate. Several studies led to the conclusion that avoiding or delaying the use of corticosteroids may result in maximizing the potential treatment benefits of immunotherapy [12–16]. However, other evidences highlight that corticosteroids have no detrimental effect on immunotherapy and high doses of steroids might reflect poorer basal conditions (e.g., active brain metastases, concurrent diseases, larger tumor volume), ultimately responsible for the more scarce outcomes observed with ICI [17, 18]. In our study, systemic administration of corticosteroids during ICI was associated with better PFS, ORR and DCB at the univariate analysis but lost any significant effect when adjusting for other clinicopathological factors. Corticosteroids prior to ICI did not show any significant effect on outcomes. We did not observe any difference when dividing steroid-receiving patients according to dose (above or below an equivalent of 30 mg of prednisone; not shown), as well. To note, in 48 out of 61 (78%) cases, systemic corticosteroids were administered to treat immune-related adverse events and in 5 (8%) further cases were administered as premedication to CT scan contrast medium. Thus, in our study corticosteroid use did not reflect a baseline unfavorable condition beyond tumor type and there was no hint that successfully treating ICI immune-mediated toxicities with corticosteroids might ultimately impair anti-PD1/PD-L1 efficacy.

Multiple evidences have highlighted so far the capability of the simple LIPI score, based on the derived neutrophile-to-lymphocyte ratio (dNLR) and LDH, to successfully predict the prognosis of patients with NSCLC treated with immunotherapy [36, 37]. LIPI score prognostic ability has been also evaluated in patients with various tumor types treated with ICI, like melanoma, bladder cancer or solid tumors harboring MSI [19, 22, 36, 38–40]. Our study confirms the capability of the LIPI score to successfully stratify patients with solid tumors treated with anti-PD1/PD-L1 in different prognostic subgroups, independently from all main clinicopathological characteristics, in a tumor-agnostic fashion, both in terms of PFS and OS. Patients with poor basal LIPI had a poor benefit from ICI, hence the evaluation of LIPI may identify a subset of patients with no or reduced benefit to anti-PD1/PD-L1 therapy. Considering the evidence available on this score, we strongly encourage its use at least for the selection of patients for clinical trials with ICI or as a stratification factor within such trials.

Noteworthy, an immunotherapy-naïve status was associated to a significantly better PFS, independently from other characteristics. Concordant recommendations regarding the opportunity to retreat patients already treated with immunotherapy do not exist. Furthermore, these patients are usually excluded from clinical trials that evaluate new ICI drugs or combinations so the evidence of activity in this setting is limited. A recent meta-analysis pooling 49 available studies showed that in patients who had previously discontinued ICI because of PD, ORR and median PFS were inferior to those of patients who had previously discontinued ICI because of toxicity (15.2% and 2.9 months vs. 44% and 13.2 months, respectively) [41]. Our findings, taken together with current literature, seems to confirm that rechallenges with ICI, at least with anti-PD1/PD-L1, should not be encouraged broadly, although in specific cases this strategy could be considered. Understanding the clinical impact of neo/adjuvant ICI in patients with relapsing metastatic disease candidate for immunotherapy will be of outmost importance considering the rapid expansion of therapeutic indications also in early-stage solid tumors [42, 43].

Importantly, administering anti-PD1/PD-L1 in earlier lines seemed to be associated with better PFS, OS and ORR at univariate analyses. Although the effect on PFS and OS might have been influenced by a potential lead time bias, it is also true that a less compromised immune system in untreated/less treated patients might favor the elicitation of more potent immune responses. At the same time, it is important to underline that treatment line lost its effect on all endpoints at multivariate analyses. Thus, this finding seems to suggest that treatment line should not be an eligibility criterion for ICI treatment.

Finally, we observed that NSCLC and GU tumors were associated with better survival and activity outcomes compared to the rest of solid malignancies included in our study. This result, for which a specific explanation cannot be provided in the context of this analysis, is somewhat confirmatory of the good sensitivity to immune-checkpoint inhibition observed in the clinical practice scenario. In fact, most ICI are currently approved for NSCLC, prostate, kidney and bladder urothelial cancer [44].

Our study presents several limitations worth noting. First, its observational nature limited any possibility of control with respect to the administered treatment or for a more homogeneous tumor site distribution or treatment line. Second, being a non-interventional trial, we could not realize any tumor biopsy for patients lacking tumor tissues. This prevented us from testing for PD-L1 protein levels and PD1 mRNA in all patients’ tumors. Additionally, there was no control arm. Finally, patients were treated in clinical trials, which means that some agents are not currently approved for the same clinical scenario. At the same time, this potential bias highlights the added value of a Clinical Trials Unit in an Oncology Department, which gives patients real therapeutic possibilities not otherwise or readily available in a pure clinical practice scenario. Despite limitations, our study comprehensively assessed all main clinicopathological characteristics considered in clinical practice. Data were prospectively collected and there was no specific selection bias related to excessively strict inclusion criteria, which is the typical Achilles' heel when generalizing clinical trial results to the “real-life” population [45, 46]. Furthermore, the sample size was in line with most phase II single arm trials.

To resume, only < 20% of patients with solid tumors obtain an objective and durable response with anti-PD1/PD-L1 ICI, with the magnitude and duration of response being directly associated with outcomes. The appropriate selection for patients more likely to achieve a durable response to ICI should be a priority. In this perspective, common clinicopathological factors seem not to be able to identify the best candidates for immunotherapy, except for immunotherapy-naïve status. Systemic corticosteroid administration for treating ICI-related adverse events is a feasible therapeutic strategy which seem not to negatively affect ICI efficacy, as well as systemic ATB administered during treatment. Importantly, none of our RT-treated patients experienced a beneficial abscopal effect, while RT detrimental effect when administered before starting ICI should be further elucidated in wider casuistries. Importantly, our study provides additional evidence to support the use of basal LIPI score and PD1 mRNA in tumor tissue at least to select patients for clinical trials with anti-PD1/PD-L1 ICI and/or as stratification factors, while PD-L1%, with a potential 10% cut-off, is a promising tumor-agnostic prognostic and predictive factor. However, it should be further validated in appropriately powered prospective studies and with the same detecting methodology, preferably CPS, potentially more generalizable than TPS (Supplementary materials).

To conclude, the selection of the best candidate to anti-PD1/PD-L1 therapy remains an unmet need. A better molecular characterization of responders and non-responders is key to identify currently elusive factors that prevent us from efficiently select patients for this therapeutic strategy. The ongoing evaluation of blood and tissue biomarkers from our Bioimmunoblood study will hopefully provide a much-needed contribution to this field.

### Supplementary Information

Below is the link to the electronic supplementary material.

Supplementary file 1 (DOCX 458 kb)

## Data Availability

The datasets generated during and/or analyzed during the current study are available from the Corresponding Authors (AP and FS) upon reasonable request.
