# Supplementary material for: Determinants of activity and efficacy of anti-PD1/PD-L1 therapy in patients with advanced solid tumors recruited in a clinical trials unit: a longitudinal prospective biomarker-based study
Source: Cancer Immunol Immunother. 2023 Jan 10;72(6):1709–23. doi: 10.1007/s00262-022-03360-9 (PMC10198872; doi:10.1007/s00262-022-03360-9)
Supplement: Supplementary file 1 — (DOCX 458 kb) [file 262_2022_3360_MOESM1_ESM.docx]

**SUMMARY**

[Supplementary methods 2](#_Toc121843842)

[Detailed study procedures 2](#_Toc121843843)

[Study endpoints and outcomes - extended 3](#_Toc121843844)

[Supplementary Methods References 5](#_Toc121843845)

[Supplementary figures 6](#_Toc121843846)

[Supplementary figure 1. Kaplan-Meier survival curves for the overall population 6](#_Toc121843847)

[Supplementary figure 2. Waterfall plots for responses to immune-checkpoint inhibitors 8](#_Toc121843848)

[Supplementary tables 9](#_Toc121843849)

[Supplementary table 1. Univariate analyses of progression-free and overall survival 9](#_Toc121843850)

[Supplementary table 2. Univariate analyses of overall response rates and durable clinical benefit 10](#_Toc121843851)

# Supplementary methods

## **Detailed study procedures**

A blood sample was collected from each patient at cycle 1 day 1 (C1D1), cycle 2 day 1 (C2D1) and at each radiological evaluation of response until progression. For the purpose of this analysis only basal samples were considered. Common blood chemistry tests were carried out, including the evaluation of albumin, LDH and standard leukocyte populations according to the Hospital Clinic of Barcelona (HCB) standard clinical practice methodologies. We collected data on albumin (g/L) and hemoglobin (Hb) (g/dL) levels due to their potential prognostic significance in solid tumors^1,2^. LDH (U/L), leukocyte (cells/mm^3^), neutrophil (cells/mm^3^) and lymphocyte (cells/mm^3^) levels were also collected and the lung immune prognostic index (LIPI) score was then calculated^3^. Treatments and follow-up procedures were decided outside of this study by patients’ oncologists according to their clinical practice or study protocol, in case of patients included in interventional clinical trials. All data were retrieved from electronic patient charts. In case of availability and explicit patient consent, archived tumor sections from the primary or the latest available metastatic lesion were collected. An expert pathologist from the HCB (ES) carried out an assessment of tumor-infiltrating lymphocytes (TILs) through the evaluation of hematoxylin and eosin (H&E)-stained formalin-fixed paraffin-embedded (FFPE) tumor sections (4-5um thick, magnification x 200-400), according to the methodology proposed by the International Immuno-Oncology Biomarkers Working Group^4^. We previously demonstrated a correlation between PD1 mRNA levels and response to anti-PD1 ICI across cancer types^5^. For the present study, we analyzed the expression of PD1 mRNA using the Nanostring® nCounter® platform in FFPE tumor samples across cancer types, as in our previous publication^5^. Methods for RNA extraction, quality assessment and gene expression analysis have been elsewhere described^5^. PD-L1 in tumor tissues was analyzed in FFPE tissue sections at the HCB as *per* clinical practice, using the mouse monoclonal antibody 22C3 (Dako) anti-PD-L1 monoclonal antibody on a Dako Autostainer, following manufacturer’s recommendations. PD-L1 immunohistochemical (IHC) expression in non small-cell lung cancer (NSCLC) was evaluated in tumor cells to obtain a tumor proportion score (TPS) identifying the % of tumor cells expressing PD-L1, as *per* clinical practice^6^. In all other tumors where PD-L1 positivity was tested (14/46 cases, 30%), the combined positive score (CPS) was used to assess PD-L1 status, as *per* clinical practice. The CPS is defined as the total number of tumor cells and immune cells stained with PD-L1, divided by the number of all viable tumor cells, then multiplied by 100^7^. It has been previously demonstrated that PD-L1 TPS and CPS are highly concordant in NSCLC^8^, therefore we jointed PD-L1 scores for the purpose of this exploratory analysis.

## **Study endpoints and outcomes - extended**

There was no prespecified sample size because of the exploratory nature of this study. The accrual was terminated after 4 years, and the clinical data cut-off was established when a minimum follow-up including at least one reassessment of the disease for every included patient was reached.

This study was designed to explore the potential impact of several clinical characteristics on progression-free survival (PFS), as primary endpoint, overall response rates (ORR), durable clinical benefit (DCB) and OS, as secondary endpoints.

The prognostic role of time-to-best response (TTBR) and duration of response (DOR) in patients achieving at least a stability of the disease was investigated. The primary features of interest were treatment line at which an anti-PD1 or PD-L1 ICI is delivered (1^st^ vs. subsequent lines), patients’ immunotherapy-naïve status (yes vs. no), the regimen type (ICI monotherapy vs. ICI-based combination), the ICI target (anti-PD1 vs anti-PD-L1), having received radiotherapy (RT), systemic antibiotics (ATB) or corticosteroids (>10mg prednisone equivalent dose) within 30 days before, or during ICI treatment, visceral disease (yes vs. no), as well as cancer type according to the following groups: NSCLC, genitourinary (GU) tumors (prostate, kidney and urothelial bladder cancer), gastrointestinal (GI) tumors (colorectal, gastric, esophageal, pancreatic cancer and colangiocarcinoma), breast cancer + gynecological tumors, other tumors (head & neck [H&N], glioblastoma [GB], melanoma, rare tumors). Visceral disease was defined by the presence of metastases in at least one of the following sites: liver, lung (excluding NSCLC), kidney (excluding primary), suprarenal glands, pancreas (excluding primary), intestines (excluding primary), central nervous system (excluding GB), or malignant ascites and/or pleural effusion. Non visceral disease was defined by the presence of metastases only in bone, skin, and/or lymphnodes.

Other secondary endpoints included a confirmation of the prognostic value of the LIPI score in terms of PFS and OS in a pan-cancer context, a characterization of TILs, PD-L1 and PD1 mRNA impact on ORR, DCB, PFS and OS in patients treated with ICI. The identification of potential prognostic or predictive blood biomarkers based on circulating immune cells is currently ongoing and will be the object of a dedicated publication. For the purpose of such analysis patients treated with ICI other than anti-PD1/PD-L1 will be considered, as well.

PFS was defined as the time from ICI treatment start to disease progression or patient’s death from any cause, whichever occurred first. OS was defined as the time from ICI treatment start to patient’s death from any cause. TTBR was defined as the time from ICI treatment start to the achievement of the best response, defined as progressive disease (PD), stable disease (SD), partial response (PR) or complete response (CR) according to the RECIST 1.1 criteria^9^. DOR was defined as the time from the achievement of the best response to the time of tumor progression or patient’s death from any cause, whichever occurred first. ORR was defined as the proportion of patients achieving CR and PR as their best response. DCB was defined as the proportion of patients alive and with either a CR, PR or SD retained at 6 months. Best responses were all defined according to RECIST 1.1 criteria^9^ and the evaluation of response for the purpose of this study were performed independently by the same expert (JGC) from the clinical trials unit of the HCB.

## **Supplementary Methods References**

1. Gupta D, Lis CG. Pretreatment serum albumin as a predictor of cancer survival: a systematic review of the epidemiological literature. *Nutr J*. December 22, 2010;9:69.

2. Caro JJ, Salas M, Ward A, Goss G. Anemia as an independent prognostic factor for survival in patients with cancer: a systemic, quantitative review. *Cancer*. June 15, 2001;91(12):2214–2221.

3. Aldea M, Benitez JC, Mezquita L. The Lung Immune Prognostic Index (LIPI) stratifies prognostic groups in advanced non-small cell lung cancer (NSCLC) patients. *Transl Lung Cancer Res*. August 2020;9(4):967–970.

4. Hendry S, Salgado R, Gevaert T, Russell PA, John T, Thapa B, et al. Assessing tumor infiltrating lymphocytes in solid tumors: a practical review for pathologists and proposal for a standardized method from the International Immuno-Oncology Biomarkers Working Group. *Adv Anat Pathol*. September 2017;24(5):235–251.

5. Paré L, Pascual T, Seguí E, Teixidó C, Gonzalez-Cao M, Galván P, et al. Association between PD1 mRNA and response to anti-PD1 monotherapy across multiple cancer types. *Ann Oncol*. October 1, 2018;29(10):2121–2128.

6. Vigliar E, Malapelle U, Iaccarino A, Acanfora G, Pisapia P, Clery E, et al. PD-L1 expression on routine samples of non-small cell lung cancer: results and critical issues from a 1-year experience of a centralised laboratory. *J Clin Pathol*. June 2019;72(6):412–417.

7. Schettini F, Corona SP, Giudici F, Strina C, Sirico M, Bernocchi O, et al. Clinical, Radiometabolic and Immunologic Effects of Olaparib in Locally Advanced Triple Negative Breast Cancer: The OLTRE Window of Opportunity Trial. *Frontiers in Oncology*. 2021;11:2496.

8. De Marchi P, Leal LF, Duval da Silva V, da Silva ECA, Cordeiro de Lima VC, Reis RM. PD-L1 expression by Tumor Proportion Score (TPS) and Combined Positive Score (CPS) are similar in non-small cell lung cancer (NSCLC). J Clin Pathol. 2021 Nov;74(11):735-740.

9. Eisenhauer EA, Therasse P, Bogaerts J, Schwartz LH, Sargent D, Ford R, et al. New response evaluation criteria in solid tumours: revised RECIST guideline (version 1.1). *Eur J Cancer*. January 2009;45(2):228–247.

# Supplementary figures

## **Supplementary figure 1. Kaplan-Meier survival curves for the overall population**

**Legend.** PFS (A), OS (B), TTBR (C) and DOR (D) Kaplan-Meier curves with their 95%CI for the overall population. PFS: median progression-free survival; OS: overall survival; TTBR: time-to-best response; DOR: duration of response; m: median; CI: confidence interval.

## **Supplementary figure 2. Waterfall plots for responses to immune-checkpoint inhibitors**

**Legend.** A: best responses in the overall population; B: best responses according to immune-naïve status; C: best responses according to tumor site; D: best responses according to treatment line; CR: complete response; PR: partial response; SD: stable disease; PD: progressive disease; NSCLC: non-small cell lung cancer; H&N: head and neck tumors; GI: gastrointestinal.

# Supplementary tables

## **Supplementary table 1. Univariate analyses of progression-free and overall survival**

| **Variables** | **PFS** | | | | **OS** | | | |
| --- | --- | --- | --- | --- | --- | --- | --- | --- |
|  | **HR** | **Inf 95%CI** | **Sup 95%CI** | ***P*** | **HR** | **Inf 95%CI** | **Sup 95%CI** | ***P*** |
| Age (continuous) | 0.99 | 0.98 | 1.01 | 0.414 | 1.00 | 0.98 | 1.02 | 0.861 |
| Sex (Male vs. Female) | 0.83 | 0.57 | 1.22 | 0.343 | 0.78 | 0.50 | 1.20 | 0.253 |
| ECOG (0-1 vs. 2-3) | 0.93 | 0.50 | 1.73 | 0.822 | 1.01 | 0.49 | 2.10 | 0.976 |
| Metastatic at diagnosis (Yes vs. No) | 0.79 | 0.55 | 1.13 | 0.199 | 1.02 | 0.67 | 1.55 | 0.927 |
| Cancer site (NSCLC reference) |  |  |  | ***0.009*** |  |  |  | ***0.027*** |
| *GI tumors* | 1.92 | 1.16 | 3.18 | ***0.011*** | 0.51 | 0.23 | 1.12 | 0.094 |
| *Melanoma + H&N + rare tumors* | 2.26 | 1.32 | 3.88 | ***0.003*** | 1.19 | 0.58 | 2.47 | 0.636 |
| *GU tumors* | 1.17 | 0.63 | 2.17 | 0.628 | 1.25 | 0.60 | 2.61 | 0.547 |
| *Breast cancer and gynecologic tumors* | 2.36 | 1.21 | 4.62 | ***0.012*** | 0.67 | 0.29 | 1.55 | 0.346 |
| ICI treatment line (1st vs. ≥2nd) | 0.64 | 0.42 | 0.98 | ***0.037*** | 0.70 | 0.43 | 1.16 | 0.164 |
| ICI treatment line (continuous) | 1.24 | 1.10 | 1.40 | ***0.001*** | 1.15 | 1.01 | 1.31 | ***0.037*** |
| Immuno-naive status (Yes vs. No) | 0.42 | 0.25 | 0.72 | ***0.001*** | 0.60 | 0.33 | 1.11 | 0.098 |
| Regimen (Monotherapy vs. Combination) | 0.92 | 0.64 | 1.32 | 0.644 | 0.83 | 0.55 | 1.26 | 0.389 |
| ICI target (Anti-PD-L1 vs. Anti-PD1) | 0.95 | 0.62 | 1.45 | 0.803 | 0.91 | 0.57 | 1.46 | 0.697 |
| Visceral disease (Yes vs. No) | 0.88 | 0.58 | 1.33 | 0.527 | 0.85 | 0.53 | 1.37 | 0.510 |
| Number of metastases (≥3 vs. <3) | 1.06 | 0.67 | 1.67 | 0.795 | 0.89 | 0.53 | 1.49 | 0.644 |
| Basal LIPI Score |  |  |  | ***0.008*** |  |  |  | ***<0.001*** |
| *Intermediate vs. Good* | 1.55 | 1.03 | 2.32 | ***0.035*** | 1.85 | 1.15 | 2.97 | ***0.011*** |
| *Intermediate vs. Poor* | 0.68 | 0.39 | 1.12 | 0.181 | 0.48 | 0.26 | 0.89 | ***0.019*** |
| *Poor vs. Good* | 2.28 | 1.29 | 4.03 | ***0.005*** | 3.89 | 2.03 | 7.42 | ***<0.001*** |
| Basal Hb (continous) | 1.00 | 0.99 | 1.01 | 0.867 | 0.99 | 0.98 | 1.01 | 0.362 |
| Basal Albumin (continous) | 0.96 | 0.92 | 1.01 | 0.092 | 0.97 | 0.92 | 1.02 | 0.270 |
| RT within 30 days from ICI start (Yes vs. No) | 1.35 | 0.66 | 2.77 | 0.412 | 2.74 | 1.24 | 6.02 | ***0.009*** |
| RT during ICI (Yes vs. No) | 1.11 | 0.73 | 1.70 | 0.625 | 1.22 | 0.77 | 1.93 | 0.405 |
| sATB within 30 days from ICI start (Yes vs. No) | 0.54 | 0.35 | 0.83 | 0.792 | 1.14 | 0.42 | 3.12 | 0.797 |
| sATB during ICI (Yes vs. No) | 0.54 | 0.35 | 0.83 | ***0.004*** | 0.63 | 0.39 | 1.03 | 0.060 |
| Corticosteroids within 30 days from ICI start (Yes vs. No) | 0.87 | 0.53 | 1.44 | 0.582 | 1.06 | 0.60 | 1.89 | 0.842 |
| Corticosteroids during ICI (Yes vs. No) | 0.58 | 0.39 | 0.85 | ***0.004*** | 0.77 | 0.50 | 1.19 | 0.241 |
| TILs % (continous) | 1.00 | 0.97 | 1.03 | 0.730 | 0.99 | 0.96 | 1.02 | 0.509 |
| PD-L1 % (continuous) | 0.99 | 0.98 | 1.00 | ***0.003*** | 0.99 | 0.98 | 1.00 | ***0.009*** |
| PD-L1 % (>10% vs. ≤10%) | 0.32 | 0.16 | 0.66 | ***0.002*** | 0.36 | 0.15 | 0.83 | ***0.016*** |
| PD1 mRNA (continuous) | 0.97 | 0.82 | 1.14 | 0.682 | 0.89 | 0.74 | 1.07 | 0.208 |
| TTBR (continuous) | - | - | - | *-* | 0.54 | 0.39 | 0.76 | ***<0.001*** |
| ORR (CR+PR vs. SD+PD) | - | - | - | *-* | 0.12 | 0.05 | 0.30 | ***<0.001*** |

**Legend.** HR: hazard ratio; inferior; Sup: superior; PFS: progression-free survival; OS: overall survival; ICI: immune-checkpoint inhibitor; TILs: tumor-infiltrating lymphocytes; TTBR: time-to-best response; ORR: overall response rates; CR: complete response; PR: partial response; SD: stable disease; PD: progressive disease; NSCLC: non-small cell lung cancer; H&N: head and neck tumors; GI: gastrointestinal; GU: genitourinary; sATB: systemic antibiotics; RT: radiotherapy.

## **Supplementary table 2. Univariate analyses of overall response rates and durable clinical benefit**

| **Variables** | **Univariate Analyses** | | | | | | | |
| --- | --- | --- | --- | --- | --- | --- | --- | --- |
|  | **ORR** | | | | **DCB** | | | |
|  | **OR** | **Inf 95%CI** | **Sup 95%CI** | **P** | **OR** | **Inf 95%CI** | **Sup 95%CI** | **P** |
| Age (continuous) | 1.02 | 0.98 | 1.05 | 0.439 | 1.02 | 0.98 | 1.06 | 0.319 |
| Sex (Male vs. Female) | 1.46 | 0.57 | 3.76 | 0.431 | 1.12 | 0.47 | 2.63 | 0.802 |
| ECOG (0-1 vs. 2-3) | 1.21 | 0.31 | 4.72 | 0.779 | 0.50 | 0.14 | 1.75 | 0.279 |
| Metastatic at diagnosis (Yes vs. No) | 1.55 | 0.64 | 3.74 | 0.335 | 1.55 | 0.64 | 3.74 | 0.335 |
| Cancer site (NSCLC+GU vs. all others) | 3.15 | 1.30 | 7.65 | ***0.011*** | 3.15 | 1.30 | 7.65 | ***0.011*** |
| ICI treatment line (1st vs. ≥2nd) | 2.82 | 1.17 | 6.79 | ***0.021*** | 1.52 | 0.62 | 3.76 | 0.365 |
| ICI treatment line (continuous) | 0.61 | 0.40 | 0.91 | ***0.016*** | 0.78 | 0.56 | 1.09 | 0.149 |
| Immuno-naive status (Yes vs. No) | 3.57 | 0.45 | 28.32 | 0.228 | 3.57 | 0.45 | 28.32 | 0.228 |
| Regimen (Monotherapy vs. Combination) | 1.47 | 0.63 | 3.45 | 0.371 | 0.69 | 0.29 | 1.65 | 0.403 |
| ICI target (Anti-PD-L1 vs. Anti-PD1) | 0.82 | 0.28 | 2.38 | 0.715 | 1.40 | 0.53 | 3.70 | 0.498 |
| Visceral disease (Yes vs. No) | 1.28 | 0.44 | 3.70 | 0.651 | 1.23 | 0.44 | 3.70 | 0.651 |
| Number of metastases (≥3 vs. <3) | 0.79 | 0.29 | 2.19 | 0.651 | 2.12 | 0.59 | 7.62 | 0.249 |
| Basal Hb (continous) | 1.01 | 0.98 | 1.03 | 0.462 | 0.98 | 0.96 | 1.01 | 0.216 |
| Basal Albumin (continous) | 1.02 | 0.92 | 1.13 | 0.729 | 1.00 | 0.91 | 1.11 | 1.00 |
| RT within 30 days from ICI start (Yes vs. No) | 0.57 | 0.07 | 4.80 | 0.607 | 0.57 | 0.07 | 4.80 | 0.607 |
| RT during ICI (Yes vs. No) | 0.93 | 0.34 | 2.54 | 0.882 | 1.19 | 0.45 | 3.14 | 0.720 |
| sATB within 30 days from ICI start (Yes vs. No) | 0.76 | 0.09 | 6.60 | 0.803 | 0.76 | 0.09 | 6.60 | 0.803 |
| sATB during ICI (Yes vs. No) | 2.15 | 0.89 | 5.19 | 0.089 | 4.83 | 1.98 | 11.77 | ***0.001*** |
| Corticosteroids within 30 days from ICI start (Yes vs. No) | 0.73 | 0.20 | 2.66 | 0.628 | 0.69 | 0.19 | 2.52 | 0.570 |
| Corticosteroids during ICI (Yes vs. No) | 2.42 | 1.03 | 5.73 | ***0.044*** | 2.94 | 1.24 | 7.01 | ***0.015*** |
| TILs % (continous) | 1.01 | 0.95 | 1.07 | 0.788 | 1.01 | 0.95 | 1.07 | 0.870 |
| PD-L1 % (continuous) | 1.03 | 1.01 | 1.05 | ***0.007*** | 1.03 | 1.00 | 1.05 | ***0.028*** |
| PD-L1 % (>10% vs. ≤10%) | 16.92 | 1.94 | 147.77 | ***0.011*** | 9.63 | 1.08 | 86.18 | ***0.043*** |
| PD1 mRNA (continuous) | 1.21 | 0.82 | 1.79 | 0.331 | 1.08 | 0.73 | 1.60 | 0.686 |

**Legend.** ORR: overall response rates; DCB: durable clinical benefit; OR: odds ratio; Inf: inferior; Sup: superior; ICI: immune-checkpoint inhibitor; NSCLC: non-small cell lung cancer; GU: genitourinary; TILs: tumor-infiltrating lymphocytes; sATB: systemic antibiotics; RT: radiotherapy.
